# Microbial Conjugated Linolenic Acid-Enriched Fermented Milk Using Lipase-Hydrolyzed Flaxseed Oil: Biochemical, Organoleptic and Storage Traits

**DOI:** 10.3390/foods13010021

**Published:** 2023-12-20

**Authors:** Ana Luiza Fontes, Lígia L. Pimentel, Maria João P. Monteiro, M. Rosário Domingues, Luis Miguel Rodríguez-Alcalá, Ana Maria Gomes

**Affiliations:** 1 Universidade Católica Portuguesa, CBQF—Centro de Biotecnologia e Química Fina—Laboratório Associado, Escola Superior de Biotecnologia, Rua Diogo Botelho 1327, 4169-005 Porto, Portugal; afontes@ucp.pt (A.L.F.); lpimentel@ucp.pt (L.L.P.); mjmonteiro@ucp.pt (M.J.P.M.); amgomes@ucp.pt (A.M.G.); 2 Laboratório Associado para a Química Verde—LAQV-REQUIMTE, Departamento de Química, Universidade de Aveiro, Campus Universitário de Santiago, 3810-193 Aveiro, Portugal; 3 Centro de Espectrometria de Massa, LAQV-REQUIMTE, Departamento de Química, Universidade de Aveiro, Campus Universitário de Santiago, 3810-193 Aveiro, Portugal; mrd@ua.pt; 4 CESAM, Departamento de Química, Universidade de Aveiro, Campus Universitário de Santiago, 3810-193 Aveiro, Portugal

**Keywords:** conjugated linolenic acid, fermented milk, *Bifidobacterium breve*, flaxseed oil

## Abstract

The bioactive conjugated linolenic acid (CLNA) can be microbiologically produced by different probiotic strains when in the presence of α-linolenic acid (α-LNA). Food matrices are a good vector, such as has been previously demonstrated with fermented milk enriched with microbial CLNA by *Bifidobacterium breve* DSM 20091 from lipase-hydrolyzed flaxseed oil. The aim of the present work was to further assess the nutritional, biochemical and organoleptic properties of the developed dairy product, as well as its storage stability throughout 28 days at 4 °C, proving its suitability for consumption. Milk lactose hydrolyzed into glucose (0.89 g/100 g) and galactose (0.88 g/100 g), which were further metabolized into lactic (0.42 g/100 g), acetic (0.44 g/100 g) and propionic (0.85 g/100 g) acids. Titratable acidity reached 0.69% and pH 4.93. Compared with the control (no CLNA), fat content was slightly higher (2.0 g/100 g). Acetic acid was the major volatile (83.32%), lacking important dairy flavor contributors, like acetaldehyde. Sensory analysis revealed predominant astringency and bitterness. No microbial concerns arose during storage, but the CLNA content increased, and some saturated fatty acids seemed to oxidize. In conclusion, the CLNA-enriched fermented milk revealed reasonable compositional properties, yet further improvements are needed for optimal consumer acceptance and a prolonged shelf-life.

## 1. Introduction

According to the World Health Organization, non-communicable diseases are responsible for 74% of global deaths each year. Cardiovascular diseases account for the most deaths (17.9 million/year), and are followed by cancer (9.3 million/year) [1]. People are aware that diet influences the onset of such diseases, so the search for healthier and innovative food products with functional properties that could prevent or counteract those conditions has been increasing in tendency in the last years. In accordance, several studies have focused on the identification of compounds in food with added-value properties, including those found among the lipid moieties. One example is conjugated linolenic acid isomers (CLNA), which have been associated with potential anti-carcinogenic, anti-obesity and anti-inflammatory effects [2], similarly to the well-characterized conjugated linoleic acid (CLA), but at lower doses [3].

The CLNA is naturally produced during dietary α-linolenic acid (α-LNA) biohydrogenation to stearic acid (C18:0) by ruminal bacteria [4]. Therefore, it is present in meat (up to 0.28 g/100 g fat) and milk (up to 0.39 g/100 g fat) of ruminants [3]. However, these amounts are not high enough to cause any beneficial effect, based on the recommended intake of an effective dose of 2–3 g/day [5]. The CLNA isomers can mainly be found in different vegetable oils, like jacaranda (up to 32.2 g/100 g FA) [6], bitter melon (up to 52.3 g/100 g FA) [7] or pomegranate (up to 80.7 g/100 g FA) [8] seed oils. Nevertheless, due to safety concerns, most of these vegetable oils cannot be commercialized for human consumption.

Strains isolated from dairy products and the human gastrointestinal tract, namely bifidobacteria, lactobacilli and propionibacteria, have shown the capacity to produce CLNA isomers when in the presence of α-LNA as a substrate [9]. For instance, among different bifidobacteria strains cultured with 0.37 mg/mL α-LNA, it was possible to attain substrate conversion percentages of up to 90.5% [10]. Therefore, some research works have studied microbial CLNA production in food products as a strategy to develop enriched sources. Vahvaselkä et al. [11] succeeded to enrich blackcurrant press residue slurries with *Propionibacterium freudenreichii* DSM 20270 in up to 0.29 mg/mL of CLNA. Moreover, walnut milk was enriched in up to 0.75 mg/mL of CLNA by *Bifidobacterium breve* CCFM68 [12]. However, in both research works, food matrices had to be previously lipase-hydrolyzed to release α-LNA bioavailable for bacteria.

Since more than 6 billion people worldwide consume dairy products, and, in the European, Oceanian and American continents, milk provides 12–14% of the dietary fat supply [13], some studies have tested the microbial production of CLNA isomers in dairy matrices [14,15]. However, in all cases, the substrate employed was pure α-LNA, which is too expensive. Edible vegetable oils rich in α-LNA turn out to be a more cost-effective alternative, and, to the best of our knowledge, no other study has developed a dairy product enriched in microbial CLNA using such an approach. Accordingly, this research team has been able to obtain a fermented milk enriched in CLNA isomers [16] by using a lipase-hydrolyzed commercial vegetable oil and the previously characterized CLNA-producing strain *Bifidobacterium breve* DSM 20091 [15]. However, as an essential further step in a new food product’s development, its nutritional, biochemical, and organoleptic characteristics must be studied. Furthermore, the assessment of product stability during shelf-life was performed, and this has been barely addressed in CLA/CLNA-enriched food type products.

Thus, the aim of the present work was to determine if the developed microbial CLNA-enriched fermented milk possesses compositional, sensory and stability properties that would be suitable for potential consumption.

## 2. Materials and Methods

### 2.1. Chemicals and Resources

Hexane, methanol and dimethylformamide were HPLC grade (VWR Chemicals, West Chester, PA, USA). Lactic acid (≥90%) was also purchased from VWR. Sulfuric acid was obtained from Honeywell Fluka (Charlotte, NC, USA), while sodium methoxide and methyl acetate were obtained from Acros Organics (Geel, Belgium). GLC-Nestlé36 FAME mix was obtained from Nu-Chek Prep, Inc. (Elysian, MN, USA) and butterfat CRM-164 (EU Commission; Brussels, Belgium) from Fedelco Inc. (Madrid, Spain). Undecanoic acid (98.0%) was obtained from Alfa Aesar (Haverhill, MA, USA) while glyceryl tritridecanoate (>99.0%) was obtained from Larodan (Solna, Sweden). Galactose (≥98%) and acetic acid (100%) were obtained from Merck (Darmstadt, Germany). Sodium hydroxide was procured from LabChem (LaborSpirit Lda, Loures, Portugal) and phenolphthalein from José M. Vaz Pereira S.A. (Santarém, Portugal). Supelco 37 FAME mix, methyl tricosanoate (≥99.0%), α-lactose monohydrate (≥99%, total lactose basis), glucose (≥99.5%), *Candida rugosa* (CRL) type VII lipase and formic (≥95%), propionic (99%) and citric (99%) acids were purchased from Sigma-Aldrich (St. Louis, MO, USA). Pasteurized semi-skimmed cows’ milk and flaxseed oil (FSO) were bought in local markets (Porto, Portugal).

### 2.2. Milk Fermentation

First, FSO was hydrolyzed with CRL and an oil emulsion prepared with 2% (*w*/*v*) Polysorbate 80 (food-grade; Sigma-Aldrich) as described in Fontes et al. [16].

*Bifidobacterium breve* DSM 20091 (DSMZ, Braunschweig, Germany) was activated as previously reported [16], whilst changing the pre-inoculum medium to skim milk (Oxoid, Hampshire, UK), supplemented with 0.05% (*w*/*v*) L-cysteine (food-grade; Sigma-Aldrich). Afterward, the strain was inoculated at 1% (*v*/*v*) in milk (100 mL) supplemented with 0.05% (*w*/*v*) cysteine (food-grade) and containing 2 mg/mL α-LNA added from stock hydrolyzed FSO emulsion. Milk devoid of α-LNA was used as control and similarly inoculated. Inoculated milk was then incubated at 37 °C for 22 h. Cultures were always grown under anaerobic conditions using gas-generating systems in a sealed container (GasPak EZ; BD, Franklin Lakes, NJ, EUA). Fermented milk samples were stored at 4 °C before further analysis.

### 2.3. Sugars and Organic Acids Analysis

About 1 g of non-fermented and fermented milk samples (*n* = 3) was dissolved in 5 mL sulfuric acid (5 mM), homogenized with Ultra-Turrax (18,000 rpm, 3 min) and centrifuged (5000 rpm, room temperature, 10 min). The supernatant was first filtered with filter paper and then through a 0.45 µm-pore size membrane (Chromafil; Macherey-Nagel, Düren, Germany) immediately before analytical analysis. Samples were analyzed by a high-performance liquid chromatography system that consisted of a pump (K-1001 HPLC pump; Knauer, Berlin, Germany), an ion exchange Aminex HPX-87H Column (300 × 7.8 mm; Bio-Rad, Hercules, CA, USA), a column oven and two detectors—refractive index (K-2301 RI detector; Knauer) to determine sugars, and UV spectrophotometry at 210 nm (K-2501 UV Detector; Knauer) to determine organic acids. Injection conditions were as follows: mobile phase—5 mM sulfuric acid; flow rate—0.6 mL/min; injection volume—20 µL; column temperature—65 °C; running time—30 min. Compounds were quantified through calibration curves of external standards: sugars (0.25–10 mg/mL)—lactose, galactose and glucose and organic acids (0.25–2.5 mg/mL)—citric acid, lactic acid, acetic acid, formic acid and propionic acid.

### 2.4. Acids Titration

About 10 g of non-fermented and fermented milk samples (*n* = 3) were diluted in 20 mL deionized water, homogenized with slight agitation and added with 4 drops of phenolphthalein (0.5%, *w*/*v*). Samples were thereafter titrated with sodium hydroxide (0.1 M) until a persistent pink tone was attained. The volume of sodium hydroxide used was registered and the percentage of lactic acid was calculated as follows:

$$Lactic\;acid\;\left(\%\right)=\frac{V_{NaOH}\left(L\right)\times M_{NaOH}\left(g/mol\right)\times M_{C3H6O3}(g/mol)}{m_{sample}\;(g)}\times 100$$

where $V_{NaOH}$ is the volume of sodium hydroxide used in the titration, $M_{NaOH}$ the molecular weight of sodium hydroxide, $M_{C3H6O3}$ the molecular weight of lactic acid and $m_{sample}$ the mass of sample assayed.

Milk samples’ pHs were also measured (Basic 20; Crison, Barcelona, Spain).

### 2.5. Nutritional Characterization

The fat and sugar contents of fermented milk (*n* = 2) were determined according to the Portuguese norms NP 1923:1987 and NP 704:1994 [17,18], respectively. Protein amount was measured by multiplying the total nitrogen content determined through the Kjeldahl method [19] by the conversion factor of 6.25. Dry residue was obtained by drying samples at 102 °C and ashes through incineration in muffle at 550 °C. Carbohydrate content was obtained by calculated difference.

### 2.6. Volatile Compounds Analysis

Volatiles were analyzed in a gas chromatograph (GC) 456-GC (Bruker, Billerica, MA, USA) coupled with a mass spectrometer (MS) detector EVOQ TQ (Bruker) and a solid-phase microextraction (SPME) system. Approximately 5 g of fermented milk samples (*n* = 2) were added to 20 mL-glass vials. Samples were first incubated at 40 °C for 5 min and then a SPME fiber (divinylbenzene/carboxen/polydimethylsiloxane, 50/30 mm; Supelco, Bellefonte, PA, USA) was inserted to absorb volatile compounds during 30 min at 40 °C with occasional agitation. Volatile compounds were thereafter desorbed into the injector for 15 min and separated by a CP-Wax 58 FFAP CB column (50 m × 0.25 mm × 0.20 µm; Agilent Technologies, Santa Clara, CA, USA) at the following conditions: injector temperature—220 °C; split—1:30 (30 s); carrying gas—helium at 1 mL/min flow; oven temperature program—40 °C held for 1 min, raised at 2 °C/min until 220 °C and held 30 min. Mass spectra were obtained by a mass range of *m*/*z* 45–350 in fullscan mode (ion source 230 °C). Volatile compounds’ identification was based on mass spectra NIST database and results were expressed as relative area percentages.

### 2.7. Sensory Analysis

Before sensory analysis, additional fermented milk samples were used for the determination of total microbial and *Enterobacteriaceae* counts, through plating of sequential dilutions in Plate Count Agar (PCA; Biokar, Allonne, France) and Violet Red Bile Glucose Agar (VRBGA; Biokar) plates, and incubation for 24 h at 30 °C or 37 °C in aerobiosis, respectively.

Once microbiological safety was guaranteed, fermented milk samples were subjected to sensory characterization by a senior trained panel (*n* = 8). Samples were assessed in terms of appearance, aroma, texture, mouthfeel, flavor and after-taste.

### 2.8. Storage Stability Assessment

Fermented milk samples were stored at 4 °C for 28 days, being air- and light-protected with parafilm and aluminum foil. Independent flasks (*n* = 3) were collected at time 0 and every 7 days until the end of the experiment for further analyses: *B. breve* viable cell numbers determination through plating on cys-MRS agar plates of sequential decimal dilutions, total microbial counts determination through plating on PCA plates of sequential decimal dilutions, pH measurement (Basic 20; Crison, Barcelona, Spain) and fatty acid (FA) analysis (Section 2.9).

### 2.9. Fatty Acids Analysis

For the FA analysis, oil emulsion (10 µL) and fermented milk (500 mg) samples were prepared according to Pimentel et al. [20], with modifications as described in Fontes et al. [16].

The recovered FAME extracts were analyzed using a gas chromatograph Agilent 8860 (Agilent Technologies), equipped with a flame-ionization detector (GLC-FID) and a BPX70 capillary column (60 m × 0.32 mm × 0.25 μm; SGE Europe Ltd., Courtaboeuf, France) at conditions previously reported by Fontes et al. [15]. Supelco 37 and FAME from CRM-164 were used for the identification of FAs. The identification of CLA and CLNA isomers was based on previous data [15,16]. GLC-Nestlé36 was assayed for calculation of response factors and detection and quantification limits (LOD: 0.79 ng FA/mL; LOQ: 2.64 ng FA/mL).

### 2.10. Statistical Analysis

Results are reported as mean values ± standard deviation of at least duplicate samples. Data were first analyzed for normality distribution. Levene’s test was applied to verify the homogeneity of the variances. Afterward, if normality was guaranteed, one-way ANOVA (equal variances) or Welch test (no equal variances) was applied to compare more than two groups, with post hoc Bonferroni (equal variances) or Games–Howell (no equal variances). If normality was not guaranteed, the comparison between two groups was carried out by applying the Mann–Whitney test, and to compare more than two groups, the Kruskal–Wallis test was performed followed by pairwise comparison with the Mann–Whitney test. Level of significance was set in general at 0.05; in terms of CFU, differences had to be >1 log10 and pH differences had to be ≥0.5 units. Analyses were performed using IBM SPSS Statistics 28 (SPSS Inc., IBM Corporation, New York, NY, USA).

## 3. Results and Discussion

### 3.1. Sugars and Organic Acids Composition, Titratable Acidity and pH

#### 3.1.1. Sugars

Non-fermented milk contained 5.34 g/100 g of lactose, which was further hydrolyzed into glucose (0.89–1.03 g/100 g) and galactose (0.88–1.02 g/100 g) in both fermented milks, decreasing lactose content (*p* < 0.05) to 3.35–3.54 g/100 g (Table 1). Moreover, no differences (*p* > 0.05) were found in the amount of lactose or its monomers between the control and the CLNA-enriched milk (Table 1).

Beta-galactosidases, also known as lactases, are responsible for lactose hydrolysis into glucose and galactose [21], and β-galactosidase activity has been reported in different species of bifidobacteria, including *B. breve* [22]. Such capacity is essential for bacterial metabolism where it uses the released glucose for further energy production.

#### 3.1.2. Organic Acids

Citric and formic acids were present in the non-fermented milk (0.92 and 0.29 g/100 g, respectively), but its contents did not differ (*p* > 0.05) from the control or CLNA-enriched fermented milks (citric acid: 0.84–0.96 g/100 g; formic acid: 0.25–0.32 g/100 g) (Table 1). This is in accordance with Nguyen et al. [23], who observed no alteration in the citric or formic acid contents in reconstituted skimmed milk after fermentation using a *B. breve* strain.

On the other hand, additional organic acids were detected in both fermented milks, namely lactic (0.37–0.42 g/100 g), acetic (0.40–0.44 g/100 g) and propionic (0.85–0.88 g/100 g) acids (Table 1). Bifidobacteria are known as heterofermentative bacteria, producing lactic and acetic acids during growth through the fructose-6-phosphate pathway [23]. The lactic acid/acetic acid ratio obtained was c.a. 0.9; such a balanced amount of acetic acid versus that of lactic acid is important towards the preservation of the sensory quality of the fermented milks. Propionic acid results from the fermentation of glucose or lactic acid [24]. To the best of our knowledge, among bifidobacteria, its production has been reported for *B. longum*, *B. bifidum*, *B. infantis* and *B. lactis* species [25,26]. In fact, no propionic acid was detected in milk fermented by *B. breve* ATCC 15700 [23]. The present research work suggests that propionic acid can eventually be produced by *B. breve* species, depending on the strain.

Furthermore, when comparing the control fermented milk with the CLNA-enriched counterpart, no differences (*p* > 0.05) were found among organic acids’ contents (Table 1).

#### 3.1.3. Titratable Acidity and pH

As expected from a fermentation process, the titratable acidity values in both fermented milks were higher (*p* < 0.05) than in those of the non-fermented milk (0.13%; Table 1). Moreover, the CLNA-enriched fermented milk titratable acidity value was significantly higher (*p* < 0.05) than that of the control (0.69% vs. 0.50%, respectively; Table 1). This difference must have resulted from the added hydrolyzed FSO in the case of the CLNA-enriched milk. The titratable acidity of typical fermented milk products is within the range of 0.7–1.1% [27,28]; however, the bacteria employed in those dairy products consist of starter cultures commercialized for such a purpose.

The pH values were accordingly lower (>0.5-unit difference) in both the fermented milks, when comparing with the non-fermented milk (pH 6.79; Table 1). Although not significant (<0.5-unit difference), the CLNA-enriched fermented milk pH was slightly lower than that of the control (pH 4.93 vs. 5.10, respectively; Table 1), correlated inversely with the titratable acidity values (lower pH associated with higher titratable acidity for CLNA-enriched fermented milk), and associated to the added hydrolyzed FSO. Fermented dairy products with low pH (i.e., 4.0–4.5) may incur low sensory acceptance [29]; thus, when developing such products, fermentation is normally interrupted by the time the food matrix reaches pH 4.5 [30,31]. As for the present study, this was not a limiting parameter, since according to previous tests, pH would not decrease to less than pH 4.86 after 22 h of fermentation.

### 3.2. Nutritional Composition

The physico-chemical composition of the developed control and CLNA-enriched fermented milk was evaluated and is listed in Table 2. All compositional parameters (protein, carbohydrates, sugars and ash contents) were similar between both fermented milks except for fat content, which was significantly higher (*p* < 0.05) in CLNA-enriched fermented milk (2.00 g/100 g vs. 1.70 g/100 g in control) (Table 2), as expected given the higher FA content resulting from the added hydrolyzed FSO and produced CLNA isomers.

A comparison with the nutritional composition of the closest product in the Portuguese market—a semi-skimmed natural yogurt [32]—showed comparable values, with 5 g/100 g of carbohydrates, 87.9 g/100 g of water and 0.75 g/100 g of minerals. The fat content of yogurt (1.8 g/100 g) was similar to that of the control fermented milk. The protein content was in line with that listed in the nutritional table of the commercial milk employed (3.4 g/100 g).

### 3.3. Volatile Compounds Composition

After the analysis of volatile compounds in the control and CLNA-enriched fermented milks, several compounds were identified: seven ketones (acetone, 2-butanone, 2,3-butanedieone, 2-heptanone, 3-octanone, acetoin and 2-nonanone), three hydrocarbons (decane, undecane and dodecane), three alcohols (2-heptanol, 1-hexanol and 2-ethyl-1-hexanol), five carboxylic acids or esters (methyl hexanoate, acetic, butanoic, hexanoic and octanoic acids) and three sulphur compounds (methyl thiolacetate, dihydro-2-methyl-3(2H)-thiophenone and dimethyl sulfone) (Table 3). Acetaldehyde, diacetyl, acetoin, acetone and 2-butanone constitute the principal compounds responsible for the typical flavor of fermented dairy products like yogurt [33]. The key compound for yogurt aroma is acetaldehyde [34]; however, this volatile flavor was not detected in any of the fermented milks. Although the acetaldehyde production potential has been reported for different *Bifidobacterium* species, revealing a species- and strain-dependent trait [35,36], it appears that *B. breve* DSM 20091 does not present such a capacity. The major volatile compound detected was acetic acid (83%; Table 3), similar to the results reported for a fermented milk using other *Bifidobacterium* strains, namely *B. animalis* subsp. *lactis* Bb-12, where ~90% of the acetic acid was detected [37].

When comparing with the control fermented milk, differences were observed in the total ketones content, which was significantly lower (*p* < 0.05) in CLNA-enriched milk (6.05% vs. 9.02%; Table 3). Ketones constitute important flavor compounds in fermented milk [38]. On the other hand, the number of hydrocarbons was higher (*p* < 0.05) in the CLNA-enriched milk (1.04% vs. 0.31%; Table 3). Nevertheless, hydrocarbons are not considered major flavor contributors due to their high threshold [39].

### 3.4. Sensory Properties

The determination of total microbial and *Enterobacteriaceae* viable cell counts revealed that both fermented milks were safe for consumption, as numbers were below the countable range (Total microbial counts <3.00 × 10^3^ CFU/mL; Coliform counts <3.00 × 10^2^ CFU/mL).

**Figure 1:**
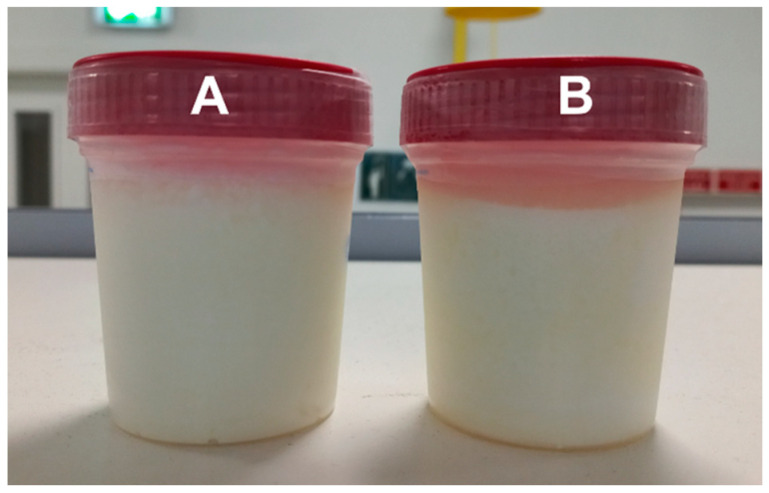
Control (**A**) and CLNA-enriched (**B**) fermented milks.

Within the sensory parameters evaluated in the CLNA-enriched fermented milk, the first assessment was the detection of a high syneresis volume (Figure 1) and a tenuous aroma of reduced sulfur compounds, while in the control fermented milk, an intense aroma of reduced sulfur compounds was perceived. Volatile sulfur compounds share common thiol precursors, such as methanethiol or hydrogen sulfide, and these thiols can arise from the biodegradation of the sulfur/carbon bound of methionine or cysteine by bacteria [40]. Probiotic bacteria belonging to the *Lactobacillus* genera showed the production of reduced sulfur compounds, such as hydrogen sulfide, methanethiol, dimethyl disulfide and dimethyl trisulfide, when in the presence of cysteine or methionine [41]. Moreover, Montoya et al. [42] observed the production of dimethyl trisulfide by a *B. breve* strain in a Swiss cheese curd slurry model system, and associated it with the free methionine naturally present. However, no reduced sulfur compounds were detected in the fermented milks (which contained added cysteine) when volatiles were analyzed, but that could be related to the very low odor threshold of such compounds [43].

Furthermore, the CLNA-enriched fermented milk also revealed an herbal aroma, reminiscent of melon peel, cucumber or aloe vera, but in the control fermented milk, a lactic aroma was distinguished, reminiscent of natural yogurt or kefir, and very slight notes of butter. The herbal flavor detected in the CLNA-enriched fermented milk must be associated with the hydrolyzed FSO added, with a higher content (*p* < 0.05) of 1-hexanol (0.71% vs. 0.29% in control; Table 3) being detected, which is characterized as a green aroma [44].

In terms of apparent texture, the CLNA-enriched fermented milk was similar to a consistent gel whereas the control fermented milk appeared as a very slight gel. Regarding mouthfeel, in the CLNA-enriched fermented milk, a cucumber flavor, high astringency, medium–high bitterness and slight acidity predominated, while in the control fermented milk, the main features that predominated were reduced sulfur compounds, low acidity and sweetness and a fine and watery texture (neither creamy nor granular). In what concerns after-taste astringency, bitterness and cucumber flavor predominated in the CLNA-enriched fermented milk, while in the control counterpart, a sulfur aroma predominated. The high astringency and bitterness of CLNA-enriched fermented milk must be related to the FSO presence.

The poor flavor properties perceived in the CLNA-enriched fermented milk must be in part associated with the fact that the *B. breve* alone was unable to generate enough lactic acid to develop a fermented dairy product with the appropriate aroma and flavor. In general, bifidobacteria are normally co-cultured with lactic acid bacterial starters, such as *Streptococcus thermophilus* and *Lactobacillus delbrueckii* ssp. *bulgaricus*, which are capable of producing suitable flavors in dairy products [45].

### 3.5. Stability during Storage

#### 3.5.1. Microbiological Enumeration and pH

*Bifidobacterium breve* DSM 20091 viable cell numbers, which were found initially (T0d) at 7.41 (control fermented milk) and 8.09 log_10_ CFU/mL (CLNA-enriched fermented milk), decreased significantly (>1 log_10_ CFU/mL difference) after 7 days of storage (T7d) at 4 °C to 5.92 and 4.51 log_10_ CFU/mL, respectively. Thereafter, viable cell numbers kept decreasing to levels below the detectable range (i.e., <5.00 × 10^3^ CFU/mL) until the end of the storage period (T28d) for both assayed conditions. According to Odamaki et al. [46], microorganisms are often exposed to several stress factors during the refrigerated storage of fermented milk, including low pH, low temperature, high osmotic pressure, nutrient starvation and oxidation, leading to a loss of viability, some of which could also justify the loss of viability observed for *B. breve* DSM 20091. Odamaki et al. [46] were able to show that *B. longum* BB536 survival in fermented milk during refrigerated storage could be improved by co-culturing with *Lactococcus lactis* subsp. *lactis* MCC866, which protected cells from oxidative stress.

As for total microbial counts, these were found to be below the detectable range (i.e., <3.00 × 10^3^ CFU/mL) at T0d and during the entire storage period at 4 °C for both fermented milks conditions. Elsewhere, low and constant counts (<2.00 log_10_ CFU/mL) of other microorganisms, like coliforms, yeasts and molds, were also observed for the control or CLNA-enriched yogurts—prepared with Pomegranate or Jacaranda seed powders—after 28 days at 4 °C [47].

Regarding the fermented milk pH, the levels maintained consistency during the whole storage period for both conditions, with values around pH 5.05–5.14 for the control and pH 4.86–4.99 for the CLNA-enriched milk. Constant pHs have also been reported for kefir [48] and yogurt [47] products during 14 and 28 days of refrigerated (4 °C) storage, respectively, whether enriched with conjugated fatty acids or not.

#### 3.5.2. Fatty Acid Profile

Regarding the FA composition in the control fermented milk, there were variations in the amount of saturated and monounsaturated FFA during refrigerated storage, with a decreasing tendency, as observed for C14 (from 0.131 to 0.106 mg/g), C16 (from 0.340 to 0.275 mg/g) and C18:1 c9 (from 0.160 to 0.142 mg/g) (Table 4) after 28 days from the beginning of storage (*p* > 0.05). On the other hand, saturated EFA decreased significantly (*p* < 0.05) throughout refrigerated storage, namely C16 (from 4.145 to 3.694 mg/g) and C18 (from 1.329 to 0.995 mg/g) (Table 5). It could be hypothesized that lipolysis led to this reduction, although no further increases were observed in the FFA fraction. In fact, the opposite seemed to occur, so it is most likely that the FFA became oxidized since these lipid species are prone to oxidation, generating hydroperoxides leading to EFA oxidation as well [49].

As for CLNA-enriched fermented milk, saturated FFA values ended up being significantly (*p* < 0.05) lower by the end of storage, especially because of the C18 reduction (from 0.334 to 0.213 mg/g) (Table 4). Moreover, there were variations in saturated EFA during refrigerated storage, but C18 decreased significantly as well (*p* < 0.05; from 1.208 to 0.956 mg/g) (Table 5). When considering all the obtained data, it is suggested that oxidation processes have occurred similar to those in the control fermented milk.

On the other hand, monounsaturated and polyunsaturated FFA increased (*p* < 0.05) throughout storage; this includes C18:1 c9 (from 0.658 to 0.765 mg/g) and the CLNA C18:3 c9t11c15 (from 0.652 to 0.929 mg/g) (Table 4). Some research works have reported the production of CLA/CLNA isomers using bacterial resting cells, including *B. breve* [50,51]. Therefore, it is most likely that, even if *B. breve* DSM 20091 cells have lost viability during storage (Section 3.5.1), their enzymatic system responsible for CLNA formation remained active, leading to the observed increase in CLNA isomers. Other studies have observed that major conjugated isomers in CLNA-enriched yogurt [47] and in commercial CLA-fortified dairy products [52] were not significantly changed after 28 days or 10 weeks at 4 °C, respectively. However, in these works, CLA/CLNA isomers were supplemented into the dairy matrices and not microbiologically produced in situ.

It has been proposed that the microbial pathway where CLA isomers are formed involves the release of further compounds, including oleic acid (i.e., C18:1 c9) [53]. Therefore, the detected increment of this former FA could be related to the above-mentioned CLNA formation.

## 4. Conclusions

The developed fermented milk enriched in microbial CLNA isomers produced by *B. breve* DSM 20091, using hydrolyzed FSO as substrate source, revealed reasonable compositional characteristics, comparable to other similar food products. As for sensory properties, it lacked important flavor contributors, and astringency and bitterness were predominant. Thus, further studies to improve these organoleptic characteristics need to be performed, namely, co-culturing with conventional yogurt or lactobacilli starter cultures and the addition of an aroma. In terms of stability during refrigerated storage, microbiological enumeration and pH brought no concerns. Moreover, CLNA isomers (the bioactive compound) increased throughout storage, most likely due to active enzymatic systems from the *B. breve* strain. On the other hand, the results suggested the occurrence of an oxidation process in some of the saturated FAs, which could have contributed to quality loss and compromised the organoleptic properties of the developed fermented milk. Therefore, the inclusion of an antioxidant in the microbial CLNA-enriched fermented milk formulation should also be addressed in the future.

## Figures and Tables

**Table 1:** Sugars and organic acids composition, titratable acidity and pH of non-fermented pasteurized semi-skimmed milk, and control and CLNA-enriched fermented milks.

Compound (g/100 g)	Non-Fermented Milk ^1,2^	Fermented Milk	
		Control ^1^	CLNA-Enriched Milk ^1,2^
**Sugars**			
Lactose	5.34 ± 0.36 ^a^	3.35 ± 0.16 ^b^	3.54 ± 0.18 ^b^
Glucose	ND	1.03 ± 0.04	0.89 ± 0.06
Galactose	ND	1.02 ± 0.04	0.88 ± 0.09
**Organic acids**			
Citric acid	0.92 ± 0.07	0.86 ± 0.06	0.84 ± 0.04
Lactic acid	ND	0.37 ± 0.03	0.42 ± 0.01
Formic acid	0.29 ± 0.05	0.25 ± 0.05	0.26 ± 0.03
Acetic acid	ND	0.40 ± 0.03	0.44 ± 0.03
Propionic acid	ND	0.88 ± 0.03	0.85 ± 0.04
Titratable acidity (%)	0.13 ± 0.01 ^c^	0.50 ± 0.01 ^b^	0.69 ± 0.01 ^a^
pH	6.79 ± 0.01 ^b^	5.10 ± 0.01 ^a^	4.93 ± 0.04 ^a^

^1^ Average values ± standard deviation (*n* = 3). Different superscript letters for significant differences within rows (*p* < 0.05). ^2^ CLNA—Conjugated linolenic acid; ND—Not detected.

**Table 2:** Physico-chemical composition of control and CLNA-enriched fermented milks.

Parameter (g/100 g)	Control ^1^	CLNA-Enriched Milk ^1,2^
Fat	1.70 ± <0.01 ^b^	2.00 ± <0.01 ^a^
Carbohydrates	4.70 ± 0.01	4.60 ± 0.01
of which Sugars	3.11 ± 0.01	2.95 ± 0.01
Protein	3.23 ± 0.01	3.06 ± 0.12
Dry residue	10.30 ± 0.01	10.42 ± 0.05
Ash	0.70 ± 0.01	0.66 ± 0.01

^1^ Average values ± standard deviation (*n* = 2). Different superscript letters for significant differences within rows (*p* < 0.05). ^2^ CLNA—Conjugated linolenic acid.

**Table 3:** Volatile composition of control and CLNA-enriched fermented milks.

Compound (Relative Area %) ^2^	Control ^1,2^	CLNA-Enriched Milk ^1,2^
Acetone	1.22 ± 0.09 ^a^	0.81 ± 0.05 ^b^
2-Butanone	1.02 ± 0.16 ^a^	0.58 ± 0.08 ^b^
Diacetyl	0.78 ± 0.07	0.68 ± 0.16
2-Heptanone	0.74 ± 0.29	0.63 ± 0.12
3-Octanone	3.97 ± 0.88 ^a^	2.40 ± 0.10 ^b^
Acetoin	1.11 ± 0.13	0.95 ± 0.16
2-Nonanone	0.18 ± 0.02	ND
Σ Ketones	9.02 ± 1.42 ^a^	6.05 ± 0.67 ^b^
Decane	0.16 ± 0.04 ^b^	0.39 ± 0.04 ^a^
Undecane	0.15 ± 0.01	0.12 ± 0.03
Dodecane	ND	0.53 ± 0.19
Σ Hydrocarbons	0.31 ± 0.11 ^b^	1.04 ± 0.26 ^a^
2-Heptanol	0.17 ± 0.04	0.14 ± 0.04
1-Hexanol	0.29 ± 0.04 ^b^	0.71 ± 0.01 ^a^
2-Ethyl-1-hexanol	0.18 ± 0.03	ND
Σ Alcohols	0.64 ± 0.11	0.85 ± 0.03
Methyl hexanoate	ND	0.53 ± 0.12
Acetic acid	83.02 ± 2.32	83.32 ± 0.77
Butyric acid	0.98 ± 0.04	0.85 ± 0.01
Hexanoic acid	1.79 ± 0.09 ^a^	1.26 ± 0.16 ^b^
Octanoic acid	0.78 ± 0.06 ^a^	0.35 ± 0.02 ^b^
Σ Carboxylic acids and esters	86.57 ± 2.25	86.31 ± 1.06
Methyl thiolacetate	0.17 ± 0.03	ND
Dihydro-2-methyl-3(2H)-thiophenone	2.13 ± 0.35	2.55 ± 0.20
Dimethyl sulfone	0.09 ± 0.01	0.06 ± 0.01
Σ Sulfur compounds	2.38 ± 0.39	2.61 ± 0.18
Σ Unk	1.09 ± 0.21 ^b^	3.14 ± 0.34 ^a^

^1^ Average values ± standard deviation (*n* = 2). Different superscript letters for significant differences within rows (*p* < 0.05). ^2^ CLNA—Conjugated linolenic acid; Unk—Unknown compounds; ND—Not detected.

**Table 4:** Free fatty acid composition of control and CLNA-enriched fermented milks during storage.

FA (mg/g) ^2^	Control ^1,2^					CLNA-Enriched Milk ^1,2^				
	T0d	T7d	T14d	T21d	T28d	T0d	T7d	T14d	T21d	T28d
C4	0.018 ± 0.002	0.015 ± <0.001	0.017 ± 0.001	0.013 ± 0.001	0.015 ± 0.002	0.013 ± 0.001	0.015 ± 0.001	0.016 ± 0.001	0.014 ± 0.002	0.012 ± 0.002
C6	0.016 ± 0.002	0.012 ± 0.001	0.015 ± <0.001	0.010 ± 0.001	0.013 ± 0.002	0.010 ± 0.001	0.013 ± <0.001	0.013 ± 0.001	0.011 ± 0.001	0.010 ± 0.001
C8	0.015 ± 0.002	0.010 ± 0.001	0.014 ± <0.001	0.008 ± 0.001	0.011 ± 0.003	0.009 ± 0.001	0.011 ± <0.001	0.012 ± 0.002	0.009 ± 0.001	0.008 ± 0.001
C10	0.039 ± 0.004	0.029 ± <0.001	0.038 ± 0.001	0.024 ± 0.001	0.031 ± 0.008	0.023 ± 0.002	0.031 ± 0.001	0.031 ± 0.003	0.024 ± 0.001	0.023 ± 0.003
C12	0.048 ± 0.006	0.037 ± <0.001	0.047 ± 0.001	0.031 ± 0.002	0.038 ± 0.009	0.030 ± 0.001	0.040 ± 0.001	0.041 ± 0.003	0.032 ± 0.001	0.030 ± 0.004
C14	0.131 ± 0.015 ^a^	0.102 ± 0.002 ^b^	0.128 ± 0.004 ^a^	0.091 ± 0.005 ^b^	0.106 ± 0.025 ^ab^	0.080 ± 0.001 ^b^	0.109 ± 0.002 ^a^	0.108 ± 0.008 ^a^	0.090 ± 0.006 ^ab^	0.082 ± 0.010 ^ab^
C14:1 c9	0.017 ± 0.001	0.015 ± <0.001	0.016 ± 0.001	0.012 ± 0.001	0.012 ± 0.002	0.012 ± <0.001	0.015 ± 0.001	0.014 ± 0.001	0.012 ± <0.001	0.010 ± 0.001
C15	0.013 ± 0.002	0.010 ± <0.001	0.013 ± 0.001	0.009 ± 0.001	0.010 ± 0.002	0.008 ± <0.001	0.011 ± <0.001	0.011 ± 0.001	0.010 ± 0.001	0.009 ± 0.001
C16	0.340 ± 0.049 ^a^	0.263 ± 0.001 ^b^	0.325 ± 0.010 ^a^	0.250 ± 0.014 ^b^	0.275 ± 0.064 ^ab^	0.373 ± 0.008 ^c^	0.496 ± 0.020 ^a^	0.482 ± 0.028 ^a^	0.441 ± 0.032 ^ab^	0.401 ± 0.023 ^bc^
C16:1 c9	0.020 ± 0.003	0.015 ± <0.001	0.018 ± <0.001	0.015 ± 0.001	0.017 ± 0.003	0.013 ± <0.001	0.019 ± 0.001	0.018 ± 0.001	0.016 ± 0.001	0.015 ± 0.001
C17	0.005 ± 0.001	0.004 ± <0.001	0.005 ± <0.001	0.004 ± <0.001	0.004 ± 0.001	0.005 ± <0.001	0.006 ± <0.001	0.006 ± <0.001	0.006 ± 0.001	0.005 ± <0.001
C18	0.104 ± 0.018	0.082 ± 0.001	0.095 ± 0.004	0.075 ± 0.006	0.073 ± 0.018	0.334 ± 0.008 ^b^	0.410 ± 0.034 ^a^	0.327 ± 0.011 ^b^	0.275 ± 0.012 ^c^	0.213 ± 0.001 ^d^
C18:1 c9	0.160 ± 0.025 ^a^	0.115 ± 0.003 ^b^	0.149 ± <0.001 ^a^	0.125 ± 0.006 ^ab^	0.142 ± 0.033 ^ab^	0.658 ± 0.029 ^b^	0.821 ± 0.062 ^a^	0.810 ± 0.043 ^a^	0.776 ± 0.009 ^a^	0.765 ± 0.007 ^a^
C18:2 c9c12 (LA)	0.025 ± 0.002	0.017 ± 0.001	0.022 ± <0.001	0.020 ± 0.001	0.025 ± 0.005	0.333 ± 0.002 ^b^	0.402 ± 0.034 ^a^	0.371 ± 0.031 ^ab^	0.396 ± 0.008 ^a^	0.389 ± 0.023 ^a^
C18:3 c9c12c15 (α-LNA)	0.003 ± <0.001	0.002 ± <0.001	0.002 ± <0.001	0.002 ± <0.001	0.003 ± <0.001	0.308 ± 0.030	0.379 ± 0.044	0.307 ± 0.065	0.366 ± 0.024	0.345 ± 0.015
C18:2 c9t11 (CLA)	0.002 ± <0.001	0.002 ± <0.001	0.002 ± <0.001	0.002 ± <0.001	0.002 ± <0.001	0.089 ± 0.021	0.103 ± 0.003	0.112 ± 0.009	0.108 ± 0.001	0.139 ± 0.024
C18:2 CLA t,t	ND	ND	ND	ND	ND	0.018 ± 0.004	0.022 ± 0.002	0.023 ± 0.002	0.023 ± 0.001	0.027 ± 0.003
C18:3 c9t11c15 (CLNA)	ND	ND	ND	ND	ND	0.652 ± 0.081 ^c^	0.736 ± 0.018 ^c^	0.756 ± 0.044 ^bc^	0.775 ± 0.002 ^b^	0.929 ± 0.013 ^a^
C18:3 t9t11c15 (CLNA)	ND	ND	ND	ND	ND	0.116 ± 0.016 ^c^	0.137 ± 0.013 ^bc^	0.137 ± 0.010 ^bc^	0.155 ± 0.004 ^ab^	0.171 ± 0.008 ^a^
SFA	0.728 ± 0.101 ^a^	0.565 ± 0.002 ^b^	0.697 ± 0.020 ^a^	0.517 ± 0.031 ^b^	0.576 ± 0.132 ^ab^	0.884 ± 0.023 ^b^	1.143 ± 0.057 ^a^	1.047 ± 0.058 ^a^	0.913 ± 0.059 ^b^	0.792 ± 0.045 ^c^
MUFA	0.197 ± 0.029 ^a^	0.145 ± 0.003 ^b^	0.183 ± 0.001 ^a^	0.152 ± 0.007 ^ab^	0.171 ± 0.039 ^ab^	0.683 ± 0.029 ^b^	0.854 ± 0.064 ^a^	0.843 ± 0.045 ^a^	0.804 ± 0.010 ^a^	0.791 ± 0.005 ^a^
PUFA	0.030 ± 0.003	0.021 ± 0.001	0.026 ± <0.001	0.023 ± 0.001	0.030 ± 0.006	1.516 ± 0.090 ^c^	1.778 ± 0.113 ^b^	1.707 ± 0.119 ^bc^	1.823 ± 0.037 ^b^	1.999 ± 0.023 ^a^
Σ FA	0.955 ± 0.133 ^a^	0.731 ± 0.002 ^b^	0.906 ± 0.020 ^a^	0.692 ± 0.039 ^b^	0.777 ± 0.177 ^ab^	3.083 ± 0.142 ^b^	3.776 ± 0.234 ^a^	3.597 ± 0.219 ^a^	3.540 ± 0.032 ^a^	3.589 ± 0.017 ^a^

^1^ Average values ± standard deviation (*n* = 3). Different superscript letters for significant differences between storage times of each fermented milk sample (*p* < 0.05). ^2^ FA—Fatty acid; T0d—Time zero days; T7d—Time seven days; T14d—Time fourteen days; T21d—Time twenty-one days; T28d—Time twenty-eight days; c—cis double bond; t—trans double bond; LA—Linoleic acid; α-LNA—alpha-Linolenic acid; CLA—Conjugated linoleic acid; CLNA—Conjugated linolenic acid; SFA—Saturated fatty acids; MUFA—Monounsaturated fatty acids; PUFA—Polyunsaturated fatty acids; Σ FA—Total fatty acids; ND—Not detected.

**Table 5:** Esterified fatty acid composition of control and CLNA-enriched fermented milks during storage.

FA (mg/g) ^2^	Control ^1,2^					CLNA-Enriched Milk ^1,2^				
	T0d	T7d	T14d	T21d	T28d	T0d	T7d	T14d	T21d	T28d
C4	0.213 ± <0.001	0.202 ± 0.010	0.197 ± 0.017	0.204 ± 0.033	0.210 ± 0.002	0.192 ± 0.007 ^ab^	0.217 ± 0.009 ^a^	0.175 ± 0.003 ^b^	0.215 ± 0.025 ^a^	0.200 ± 0.006 ^a^
C6	0.180 ± 0.006	0.159 ± 0.014	0.163 ± 0.002	0.168 ± 0.019	0.165 ± 0.006	0.161 ± 0.011 ^abc^	0.182 ± 0.013 ^a^	0.144 ± 0.001 ^c^	0.177 ± 0.022 ^ab^	0.158 ± <0.001 ^bc^
C8	0.113 ± 0.010 ^a^	0.103 ± 0.007 ^ab^	0.088 ± 0.001 ^b^	0.105 ± 0.013 ^ab^	0.101 ± 0.001 ^ab^	0.096 ± 0.007	0.112 ± 0.010	0.083 ± 0.010	0.106 ± 0.011	0.102 ± 0.003
C10	0.362 ± 0.013	0.309 ± 0.034	0.337 ± 0.010	0.326 ± 0.042	0.339 ± 0.001	0.304 ± 0.008	0.329 ± 0.014	0.301 ± 0.013	0.342 ± 0.036	0.317 ± 0.007
C12	0.459 ± 0.013	0.404 ± 0.037	0.419 ± 0.011	0.409 ± 0.046	0.419 ± 0.001	0.396 ± 0.009 ^b^	0.439 ± 0.024 ^a^	0.374 ± 0.018 ^b^	0.426 ± 0.049 ^ab^	0.401 ± 0.003 ^b^
C14	1.447 ± 0.045	1.275 ± 0.109	1.307 ± 0.034	1.255 ± 0.146	1.302 ± 0.001	1.257 ± 0.021 ^b^	1.376 ± 0.074 ^a^	1.168 ± 0.044 ^b^	1.314 ± 0.152 ^ab^	1.239 ± 0.001 ^b^
C14:1 c9	0.132 ± 0.006	0.120 ± 0.009	0.125 ± 0.007	0.120 ± 0.015	0.127 ± 0.002	0.118 ± 0.001	0.125 ± 0.007	0.109 ± 0.004	0.127 ± 0.016	0.118 ± <0.001
C15	0.149 ± 0.002	0.130 ± 0.011	0.135 ± 0.003	0.128 ± 0.015	0.131 ± 0.001	0.130 ± 0.004	0.140 ± 0.010	0.120 ± 0.004	0.134 ± 0.015	0.125 ± 0.001
C16	4.145 ± 0.133 ^a^	3.623 ± 0.329 ^b^	3.743 ± 0.111 ^b^	3.562 ± 0.417 ^b^	3.694 ± 0.018 ^b^	3.632 ± 0.053 ^ab^	3.925 ± 0.203 ^a^	3.343 ± 0.118 ^b^	3.743 ± 0.437 ^ab^	3.531 ± 0.025 ^b^
C16:1 c9	0.200 ± 0.004	0.173 ± 0.015	0.188 ± 0.012	0.183 ± 0.020	0.201 ± 0.006	0.183 ± 0.024	0.184 ± 0.013	0.164 ± 0.009	0.196 ± 0.021	0.187 ± 0.004
C17	0.064 ± 0.003	0.055 ± 0.006	0.057 ± 0.003	0.052 ± 0.006	0.056 ± 0.002	0.057 ± <0.001	0.060 ± 0.003	0.051 ± 0.001	0.055 ± 0.005	0.054 ± <0.001
C18	1.329 ± 0.043 ^a^	1.170 ± 0.118 ^ab^	1.135 ± 0.044 ^b^	1.051 ± 0.125 ^bc^	0.995 ± 0.002 ^c^	1.208 ± 0.018 ^a^	1.290 ± 0.058 ^a^	1.035 ± 0.030 ^b^	1.100 ± 0.128 ^ab^	0.956 ± 0.022 ^b^
C18:1 c9	1.970 ± 0.076	1.712 ± 0.179	1.840 ± 0.060	1.806 ± 0.187	1.936 ± 0.014	1.873 ± 0.025	1.995 ± 0.103	1.766 ± 0.081	2.027 ± 0.249	1.999 ± 0.020
C18:2 c9c12 (LA)	0.268 ± 0.010 ^ab^	0.226 ± 0.024 ^b^	0.246 ± 0.008 ^b^	0.252 ± 0.019 ^b^	0.288 ± 0.004 ^a^	0.258 ± 0.002 ^b^	0.269 ± 0.011 ^b^	0.245 ± 0.009 ^b^	0.289 ± 0.037 ^ab^	0.299 ± 0.006 ^a^
C18:3 c9c12c15 (α-LNA)	0.029 ± 0.001	0.025 ± 0.004	0.027 ± 0.001	0.025 ± 0.004	0.032 ± 0.001	0.107 ± 0.003 ^a^	0.091 ± 0.004 ^ab^	0.079 ± 0.009 ^b^	0.093 ± 0.020 ^ab^	0.105 ± 0.005 ^a^
C18:2 c9t11 (CLA)	0.027 ± 0.003	0.023 ± 0.002	0.025 ± 0.004	0.027 ± 0.002	0.035 ± 0.001	0.028 ± 0.002	0.026 ± 0.001	0.027 ± 0.001	0.032 ± 0.003	0.037 ± 0.001
SFA	8.461 ± 0.266 ^a^	7.430 ± 0.672 ^b^	7.582 ± 0.223 ^b^	7.260 ± 0.863 ^b^	7.412 ± 0.016 ^b^	7.433 ± 0.121 ^b^	8.070 ± 0.418 ^a^	6.795 ± 0.242 ^b^	7.612 ± 0.876 ^ab^	7.082 ± 0.068 ^b^
MUFA	2.302 ± 0.084 ^a^	2.004 ± 0.202 ^b^	2.153 ± 0.079 ^ab^	2.109 ± 0.223 ^ab^	2.264 ± 0.021 ^a^	2.175 ± 0.048	2.304 ± 0.123	2.040 ± 0.093	2.351 ± 0.286	2.305 ± 0.016
PUFA	0.324 ± 0.013 ^ab^	0.274 ± 0.030 ^b^	0.298 ± 0.013 ^b^	0.304 ± 0.025 ^b^	0.355 ± 0.006 ^a^	0.394 ± 0.004 ^b^	0.387 ± 0.016 ^b^	0.350 ± 0.020 ^b^	0.414 ± 0.058 ^ab^	0.440 ± 0.002 ^a^
Σ FA	11.086 ± 0.363	9.708 ± 0.904	10.033 ± 0.315	9.673 ± 1.110	10.031 ± 0.031	10.002 ± 0.173 ^b^	10.761 ± 0.557 ^a^	9.184 ± 0.355 ^b^	10.377 ± 1.217 ^ab^	9.826 ± 0.086 ^b^

^1^ Average values ± standard deviation (*n* = 3). Different superscript letters for significant differences between storage times of each fermented milk sample (*p* < 0.05). ^2^ FA—Fatty acid; T0d—Time zero days; T7d—Time seven days; T14d—Time fourteen days; T21d—Time twenty-one days; T28d—Time twenty-eight days; c—cis double bond; t—trans double bond; LA—Linoleic acid; α-LNA—alpha-Linolenic acid; CLA—Conjugated linoleic acid; CLNA – Conjugated linolenic acid; SFA—Saturated fatty acids; MUFA—Monounsaturated fatty acids; PUFA—Polyunsaturated fatty acids; Σ FA—Total fatty acids.

## Data Availability

Data is contained within the article.
